# Cardiac aorta-derived extracellular matrix scaffold enhances critical mediators of angiogenesis in isoproterenol-induced myocardial infarction mice

**DOI:** 10.1007/s10856-021-06611-w

**Published:** 2021-10-26

**Authors:** Mahara Hosseinabadi, Zohreh Abdolmaleki, Seyed Hamed Shirazi Beheshtiha

**Affiliations:** 1 Department of Pharmacology, Karaj Branch, Islamic Azad University, Karaj, Iran; 2 Department of Clinical Sciences, Karaj Branch, Islamic Azad University, Karaj, Iran

## Abstract

An incapability to improve lost cardiac muscle caused by acute ischemic injury remains the most important deficiency of current treatments to prevent heart failure. We investigated whether cardiomyocytes culturing on cardiac aorta-derived extracellular matrix scaffold has advantageous effects on cardiomyocytes survival and angiogenesis biomarkers’ expression. Ten male NMRI mice were randomly divided into two groups: (1) control (healthy mice) and (2) myocardial infarction (MI)-induced model group (Isoproterenol/subcutaneously injection/single dose of 85 mg/kg). Two days after isoproterenol injection, all animals were sacrificed to isolate cardiomyocytes from myocardium tissues. The fresh thoracic aorta was obtained from male NMRI mice and decellularized using 4% sodium deoxycholate and 2000 kU DNase-I treatments. Control and MI-derived cardiomyocytes were seeded on decellularized cardiac aorta (DCA) considered three-dimensional (3D) cultures. To compare, the isolated cardiomyocytes from control and MI groups were also cultured as a two-dimensional (2D) culture system for 14 days. The cell viability was examined by MTT assay. The expression levels of *Hif-1α* and *VEGF* genes and VEGFR1 protein were tested by real-time PCR and western blotting, respectively. Moreover, the amount of VEGF protein was evaluated in the conditional media of the 2D and 3D systems. The oxidative stress was assessed via MDA assay. *Hif-1α* and *VEGF* genes were downregulated in MI groups compared to controls. However, the resulting data showed that decellularized cardiac aorta matrices positively affect the expression of *Hif-1α* and *VEGF* genes. The expression level of VEGFR1 protein was significantly (*p* ≤ 0.01) upregulated in both MI and healthy cell groups cultured on decellularized cardiac aorta matrices as a 3D system compared to the MI cell group cultured in the 2D systems. Furthermore, MDA concentration significantly decreased in 3D-cultured cells (MI and healthy cell groups) rather than the 2D-cultured MI group (*p* ≤ 0.015). The findings suggest that cardiac aorta-derived extracellular scaffold by preserving VEGF, improving the cell viability, and stimulating angiogenesis via upregulating *Hif-1α, VEGF*, and VEGFR1 in cardiomyocytes could be considered as a potential approach along with another therapeutic method to reduce the complications of myocardial infarction and control the progressive pathological conditions related to MI.

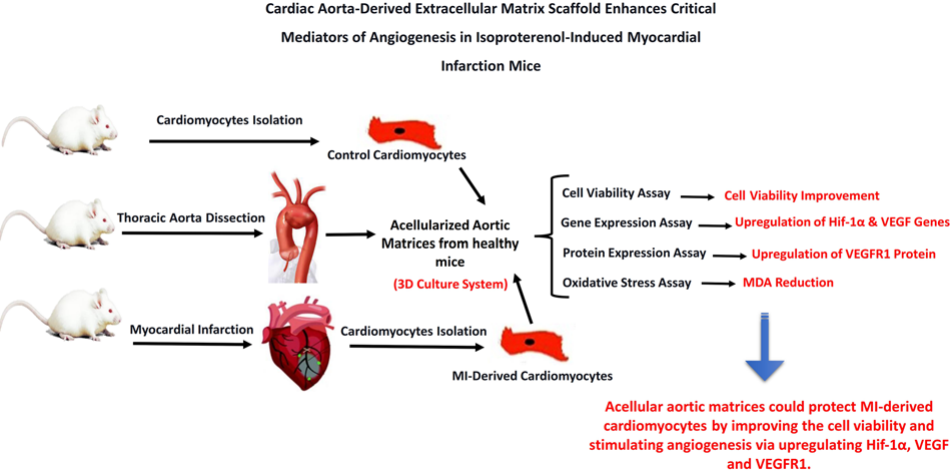

## Introduction

Myocardial infarction (MI) provoked by a coronary blockage may result in heart failure and cause cardiovascular-related mortality [1–4]. Effective treatments following myocardial infarction are needed to enhance cardiac regeneration, reduce scar formation, and improve ventricular remodeling. Tissue-derived extracellular matrix (ECM) supports cell survival, function, and adaptive tissue repair processes by providing a critical microenvironment. The ECM is a passive structural architecture that, in addition to biomechanical impacts on the cells, provides a reservoir of matricellular proteins and bioactive growth factors that influence cell phenotype and behavior [5, 6]. After an ischemic injury, dysregulation of the ECM can mediate maladaptive structural remodeling and cause a deterioration of function [7]. Therefore, tissue-derived ECM scaffolds can be a good choice for promoting adaptive healing and functional recovery after ischemic injury.

Acellular matrices/decellularized tissues can be in vitro generated from cellular sections of different tissue, using various physical and chemical agents [8]. These biomaterials lacking immunogenic issues have a structurally organized ECM comprising angiogenic growth factors, such as vascular endothelial growth factor (VEGF) and basic fibroblast growth factor [9, 10]. The functionality of the decellularized tissues obtained from canine carotid arteries, small intestinal submucosa, and porcine carotid artery has been evaluated by implanting them as xenografts in animals [11, 12].

Angiogenesis presents a critical function in MI therapy by improving myocardial regeneration, preventing heart failure, and improving cardiac function. VEGF and its receptor (VEGFR) are essential regulators of the development, differentiation, and growth of blood vessels and heart and signified by the improvement of heart structure and function [13–15]. The level of VEGF is raised in the sera of acute myocardial infarction patients [16], and it protects cardiac function post-infarction [13]. Besides, intramyocardial *VEGF* expression improves cardiac function after permanent coronary artery occlusion via overexpression of the genes that provoke the compensatory hypertrophic response. Cell therapy and growth factor’s gene delivery (e.g., *VEGF* gene) has been proved to improve the survival, engraftment, and differentiation of donor cells [17, 18]. Hif-1α, Hypoxia-inducible factor-1α, is a hypoxia environment’s main regulatory factor that responds to chronic hypoxia and is an important factor in regulating VEGF and VEGFR and other angiogenic factors [19].

This study aimed to investigate the growth, proliferation, and angiogenesis signaling pathways in cardiomyocytes derived from isoproterenol-induced MI mice, cultured on an acellular aortic matrix (as 3D culture system) and compare them to healthy and injured cells cultured in cell culture plates (as 2D culture system) containing supplemented culture medium without acellular aortic matrix. Consequently, as the first step, the acellular matrix was prepared from mice cardiac aorta, and the growth of cardiomyocytes on it was verified in vitro and the cardiomyocytes viability cultured on the 2D and 3D systems was assessed using by MTT assay. Then, the expression levels of *Hif-1α* and *VEGF* genes and VEGFR1 protein were respectively analyzed using real-time PCR and western blotting. The MDA levels as an oxidative stress marker were also evaluated for each group.

## Materials and methods

### Reagents

Isoproterenol was purchased from Sigma-Aldrich (St Louis, MO, USA). MDA kits were purchased from Sigma-Aldrich. TRIzol^®^ for total RNA extraction was purchased from Gibco (Thermo Fisher Scientific, Inc., Waltham, MA, USA). PrimeScriptTMRT Reagent Ki and SYBR^®^ Premix Ex TaqTM were purchased from TaKaRa (Shiga, Japan). The antibody against VEGFR1 was purchased from Abcam (Cambridge, MA, USA).

### Ethical aspects

This project was approved by the Ethical Committee of Islamic Azad University, Karaj, Iran (ethical code: IR.IAU.K.REC.1399.052). We also carefully follow the national and internationally accepted guidelines for the care and use of animals.

### Animals and experimental design

Ten male NMRI mice with a weight between 25 and 30 g were kept in cages under controlled ambient temperature (22 ± 2 °C) and controlled 12:12 h light-dark cycle. The mice were randomly divided into two groups (1) Control (healthy mice) and (2) MI model group (Isoproterenol injection). DL-Isoproterenol (ISO; Sigma), a nonselective beta-adrenergic agonist, was administered subcutaneously at a single dose of 85 mg/kg body weight diluted in 2 ml of saline. Animals were sacrificed 2 days after the post- ISO injection. Furthermore, fifteen healthy NMRI mice were used for aorta isolation.

### Preparation of acellular aortic matrices (AAMs)

Fifteen healthy male NMRI mice were sacrificed by Ketamine/Xylazine-induced anesthesia (50 mg/kg ketamine, 5 mg/kg xylazine). The thoracic aorta was isolated and excised from the animal body according to a comprehensive protocol provided by Robbins et al. [20]. The fresh isolated thoracic aorta was flushed twice with the phosphate-buffered solution (PBS, Merck, Germany) containing amphotericin B and antibiotics. Acellular matrices (AMs) were prepared according to Meezan et al. method [21]. Briefly, the aorta was processed with distilled water for 18 h at 4 °C and 4% sodium deoxycholate (Sigma, UK) for 4 h, and 2000 kU deoxyribonucleases I (DNase-I, Sigma, USA) in 1 M NaCl (Merck, Germany) for 3 h. The treatment was repeated twice until the cells were completely removed. Acellular matrices were stored in PBS at 4 °C until used.

### DAPI staining

To confirm the complete removal of cells from the isolated thoracic aorta tissues, DAPI nuclear staining was performed. To do this, the isolated acellular aortic tissues were fixed by 4% paraformaldehyde for 10 min. After washing with PBS, the tissues were stained with DAPI solution (0.5 µg/ml) (Thermofisher, USA) for 2 min in the dark. The samples were finally visualized by fluorescent microscope (Nikon, Japan).

### Preparation of the cells

To isolate cardiomyocytes, both healthy and MI model mice were sacrificed under anesthesia (50 mg/kg ketamine, 5 mg/kg xylazine) and then the hearts were dissected and perfused with (PBS) comprising heparin sodium (Heparodic, Iran) to rinse out the blood. To do this, a 30 gauge needle was inserted into the aorta in the left ventricle, and immediately the right ventricle was cut to allow the blood to flow out. Hearts were finely minced then placed into 10 mL digestion media (Dulbecco’s Modified Eagle’s Medium [DMEM], 100 U/mL collagenase I) and incubated at 37 °C for 40 min. The minced tissue was centrifuged at 1000 × *g* for 20 min at 4 °C. The cell pellet was solved in 2 mL of fresh digestion media and incubated at 37 °C for 20 min. The cell was diluted with 5 mL of culture media (DMEM, 10% Fetal Bovine Serum, 1% penicillin/streptomycin). The cell suspension was centrifuged at 1000 × *g* for 20 min at 4 °C. The cell pellet was suspended in 5 mL culture media and plated into T25 culture flasks (5 mL medium per flask). The cells were allowed to attach under standard culture conditions (37 °C, 5% CO_2_, 100% humidity) for 2 h, then non-adherent cells were removed by washing with PBS, and the culture medium was replaced. The culture media were changed every 2–3 days. Cardiomyocyte cultures were typically reached to proper confluence in 4–7 days. Then, cell cultures were passaged once a week.

### Cultures of myocardial cells on the acellular aortic matrices (AAMs)

For experimental purposes, the acellular aortic matrix (AAM) specimens were divided into 1 × 1 cm^2^ pieces, put into 24-well cell culture dishes, washed with PBS twice, and later incubated in DMEM media at 37 °C and 5% carbon dioxide for 10 min, before the cell seeding process. Isolated cardiomyocytes (at a density of 5 × 10^4^ cells/well) were seeded onto the luminal surface of AAMs and maintained for 14 days in the DMEM medium at 37 °C in 5% CO_2_. Also, cells were seeded in 24-well plates containing completed medium without AAMs (referred to as 2D culture system). Therefore, four-cell groups were designed to study, including (1) Healthy heart-derived cells cultured as 2D culture system (control cells), (2) MI-derived cells cultured as 2D culture system (MI cells), (3) MI-derived cells cultured on the acellular aortic matrix as 3D culture system (acellular aorta + MI cells), and (4) healthy heart-derived cells cultured on the acellular aortic matrix as 3D culture system (acellular aorta + normal cells).

### Cell viability assay

For the cell viability assay, myocytes isolated from healthy and MI model heart of mice were seeded at a density of 1 × 10^4^ cells/well in 96-well plates with (3D culture) and without (2D culture) AAMs and then were incubated for 3 and 14 days. The cell viability was evaluated using by MTT (3-(4,5-dimethylthiazol-2-yl)-2,5-diphenyltetrazolium bromide) assay. MTT solution at 5 mg/ml was added and incubated for 4 h at 37 °C. Next, the contents of all wells were discarded and 200 μl DMSO was added to each well. The absorbance of each well was recorded at a wavelength of 570 nm using an ELISA-reader. The viability percent relative to the control values were measured and calculated for each group. Each experiment was conducted in triplicate to confirm the obtained values.

### Molecular studies using real-time PCR

The gene sequences from *Hif-1α*, *VEGF*, and *β-actin* were obtained from the NCBI database, and the sequence of their exons and introns was determined to design the primers used in Real-Time PCR. Primer design was performed using the Gene runner software. Then, designed primers were blasted to verify their accuracy and reproduce only the genes’ mRNA sequences. The sequences of the Real-Time PCR primers were forward TCAGAGCAAGAGAGGCATCC and reverse GGTCATCTTCTCACGGTTGG for *β-actin*, forward CCTGCACTGAATCAAGAGGTTGC and reverse CCATCAGAAGGACTTGCTGGCT for *Hif-1α* and forward TCTCAAGTGCAGAGGGGAGG and reverse TCGAAGTAGATGTAGGGAGGT for *VEGF*. Total RNA was extracted from the two-dimensional (2D) and three-dimensional (3D) (on day 14) cultured cells by using RNX-Plus™ (Cinnagen, Iran) according to the manufacturer’s recommendations. The RNA concentration and quality were then determined using a NanoDrop 2000c (Eppendorf, Germany). cDNA was synthesized from a 1000 ng DNase-treated RNA sample with a Revert Aid™ first-strand cDNA synthesis kit (Fermentase, Lithuania) using Oligo (dT) primers. PCR was performed using Master Mix and SYBR^®^ Green (Applied Biosystems, Life Technologies, Paisley, United Kingdom) in StepOne™ Applied Biosystems according to the manufacturer’s instructions. The primers’ efficiency and specificity, the fidelity of qPCR, and melting curve analysis were determined as before. Thermocycler conditions included an initial step at 95 °C for 15 min, followed by 40 cycles at 94 °C: 20 s, 58–60 °C: 40 s, and 72 °C: 30 s. The β-actin gene was chosen as internal control against which mRNA expression of the target gene was normalized. A reference gene was used to assure the validity of the housekeeping gene and results. The resultant gene expression level was presented as 2^−ΔΔCt^, in which ΔCt was the difference between Ct values of the target gene and reference gene [22].

### Western blotting analysis

On days 3 and 14, the cells cultured in 2D or 3D conditions were lysed in RIPA (Sigma, UK) for 30 min, and total proteins were obtained. The total protein concentration was examined by Bradford assay utilizing bovine serum albumin as the standard before progressing with the western blot. At first, the protein extracts (30 µg/lane) were electrophoretically isolated on a 10% SDS-PAGE and then transferred onto a polyvinylidene difluoride membrane (EMD Millipore, Billerica, MA, USA). The membrane was then blocked using blocking buffer (phosphate-buffered saline, PBS, containing 5% non-fat dry milk) overnight at 4 °C. The proteins were probed with mouse monoclonal antibodies against VEGF and VEGFR1 (1:1000 dilution) (Abcam, UK). The membranes were incubated with goat anti-mouse HRP-conjugated secondary antibodies VEGFR1 primary antibodies (1:1000 dilution) (Abcam, UK) against VEGF and at room temperature for 2 h. The bound proteins were recognized using chemiluminescence by enhanced electrochemiluminescence reagents. Ultimately, the analysis of protein bands quantification and densitometry was performed employing ImageJ software (National Institutes of Health, imagej.nih.gov/ij).

### Measurement of malondialdehyde (MDA) activity

MDA is secondary lipid peroxidation product and can be examined with thiobarbituric acid (TBA) that can create a pink-colored adduct with the highest absorbance at 532 nm. The MDA was detected using the method described by Schmedes et al. with a minor modification [23].

Concisely, 0.2 ml cell lysate, 1.5 ml 20% acetic acid (adjusted to pH 3.5), 1.5 ml 0.9% TBA, 0.2 ml 8.1% sodium dodecyl sulfate, and 0.6 ml distilled water were vortex mixed, and the mixture was incubated in a water bath at 95 °C for 50 min. Following cooling down to 25 °C, 5.0 ml butanol: pyridine mixture and 1.0 ml of distilled water (1:15; v/v) were added. After centrifugation at 3000 rpm for 10 min, the absorbance was measured at 532 nm. The MDA concentration was calculated through a molar extinction coefficient of 1.56 × 105 M^−1^ cm^−1^, and values were shown as μmol of MDA. The standard sample was a breakdown product of 1, 1, 3, 3-tetra ethoxy propane.

### Statistical analysis

Data were examined utilizing SPSS 23.0 statistical software. One-way analysis of variance followed by the post hoc (Tukey) test was used to compare differences among all groups. Data were represented as mean ± SD. Values were considered statistically significant if the *p* values were less than 0.05.

## Results

### Cell-free confirmation of the prepared acellular aorta matrices

**Fig. 1:**
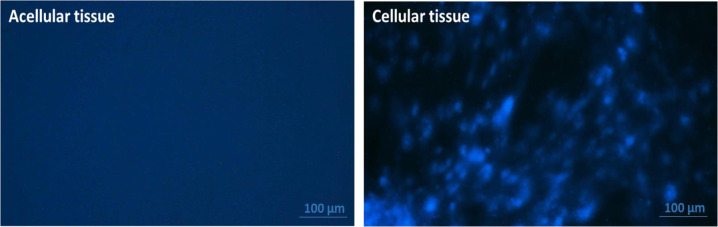
DAPI staining of the thoracic aorta tissues. DAPI fluorescent staining of the cellular and acellular aorta. The obtained images confirmed the absence of nuclei in the prepared acellular aorta matrices. Scale bar: 100 µm

As shown in Fig. 1, the fluorescent images obtained from DAPI staining showed no nuclei in the prepared aorta matrices in comparison to the cellular aorta, confirming the complete removal of cells from the decellularized tissues (Fig. 1).

### Cell proliferation assay

**Fig. 2:**
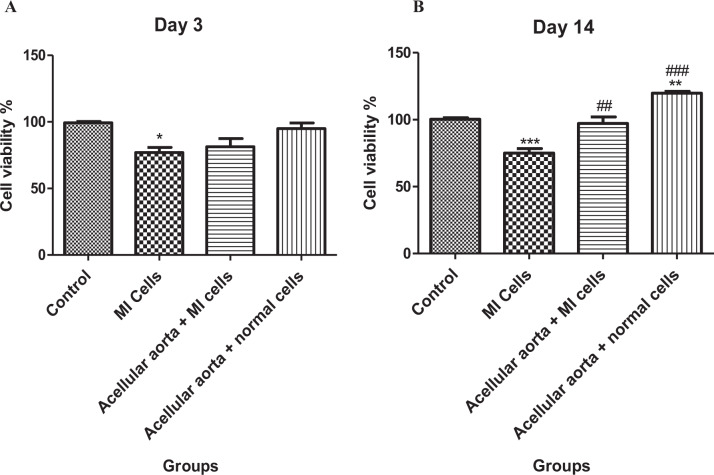
MTT assay results on cell proliferation of 3D (acellular scaffold) and 2D cultured cells. **A** MTT assays were performed on the third day. **B** MTT assays were performed on the fourteenth day. Healthy cells and MI cells were grown in 3D culture compared to their respective 2D culture were measured at days 3 and 14. Control/Healthy (normal) cells: cardiomyocytes isolated from healthy NMRI mice. MI cells: cardiomyocytes isolated from myocardial infarction NMRI mice. The obtained values are demonstrated as the means and standard deviation of the mean (mean ± SD). *Shows the significant change in comparison to control (*p* < 0.05). #shows the significant change in comparison to the MI group (*p* < 0.05). *p < 0.05, **p ≤ 0.01, and ***p ≤ 0.0001. ^#^*p* < 0.05, ^##^*p* ≤ 0.01, and ^###^*p* ≤ 0.0001

The present study examined the number of cells in the cardiomyocytes cultured under 2D and 3D conditions on days 3 and 14 by MTT assay. The results of MTT assays performed on the third day demonstrated that the proliferation rate in 2D-cultured MI cells was significantly lower compared to the control group (2D cultures of healthy cells) (Fig. 2A). There was no meaningful difference between other groups (3D-cultured healthy and MI-induced cells). However, MTT assays on the fourteenth day revealed that both healthy and MI-induced cells cultured on the acellular aorta system (3D culture) proliferate faster in comparison to cells cultured as 2D monolayer cultures (Fig. 2B). Statistically significant differences were observed on 2D-cultured MI cells and acellular aorta-cultured MI cells (*p* < 0.01). Also, there was no statistically significant difference between the control group (2D cultures of healthy cells) and the acellular aorta-cultured MI cells group (3D cultures of MI cells). There was a significant rise in the cell proliferation rate of the acellular aorta-cultured healthy cells group (3D culture) compared to 2D-cultured healthy cells (control group).

### Changes in *Hif-1α* and *VEGF* genes expression in 3D scaffold-cultured cells

**Fig. 3:**
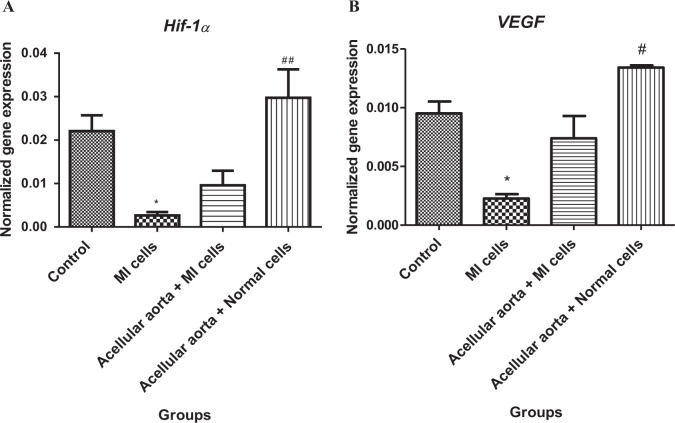
The evaluation of alterations in genes expression levels of *Hif-1α* (**A**) and *VEGF* (**B**) as antigenic factors. The quantitative data showed that the expression levels of *Hif-1α* and *VEGF* genes were severely dropped in the MI group. The acellular aorta matrix led to a remarkable increase in *Hif-1α* and *VEGF* expressions compared to the MI group. Data were statistically analyzed by one way ANOVA test, and *p* < 0.05 was considered significant. *Shows the significant change in comparison to control (*p* < 0.05). ^#^ shows the significant change in comparison to the MI group (*p* < 0.05). *p < 0.05, **p ≤ 0.01, and ***p ≤ 0.0001, ^#^*p* < 0.05, ^##^*p* ≤ 0.01, and ^###^*p* ≤ 0.0001

Given the alteration in the expression of genes involved in cardiac angiogenesis in myocardial infarction, the expression level of *Hif-1α* and *VEGF* as two critical genes in angiogenesis was evaluated by qPCR. As depicted in Fig. 3A and B, the quantitative expression data showed that *Hif-1α* and *VEGF* expression were severely reduced in 2D-cultured MI cells compared to the control group. The expression of *Hif-1α* and *VEGF* genes were significantly upregulated in acellular aorta + healthy cell groups compared to MI groups. Also, the level of *Hif-1α* and *VEGF* were increased in MI cells cultured in acellular aorta matrix compared to the MI group but not significantly. No statistically significant change in *Hif-1α* and *VEGF* expression levels was observed in healthy and MI 3D-cell culture groups compared to control. Consequently, as a result, the acellular aorta matrix could effectively lead to a notable enhancement in *Hif-1α* and *VEGF* expression.

### Protein expression of VEGFR1 and VEGF by western blot

**Fig. 4:**
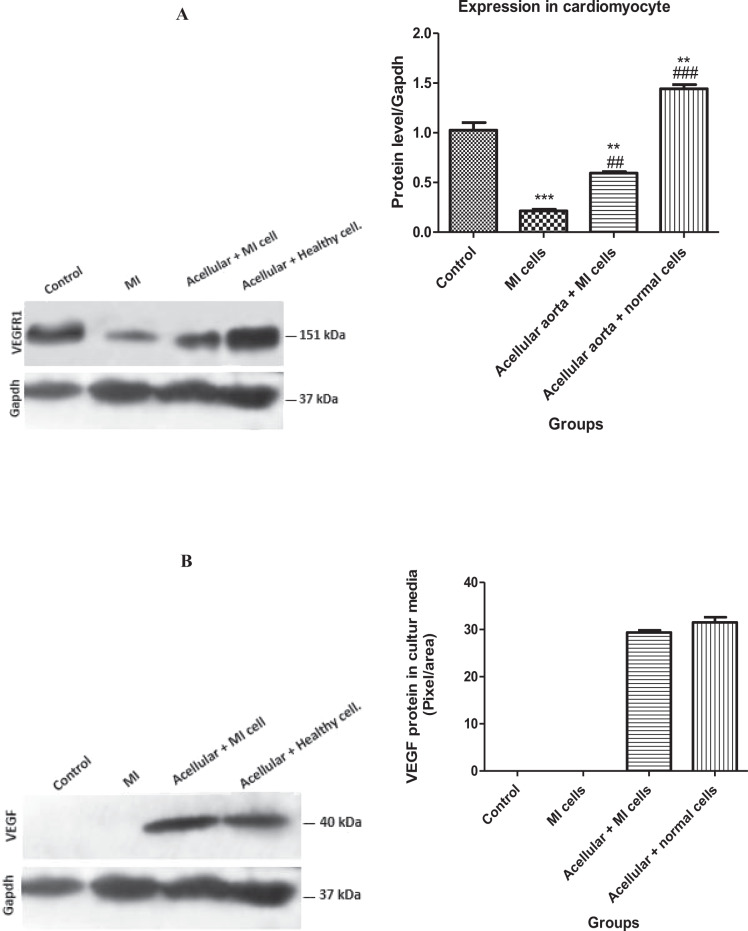
The evaluation of VEGFR1 protein expression in healthy and MI Cell lysates from 2D and 3D cultures (**A**) and VEGF protein conditioned media from 2D and 3D cultures (**B**). The VEGFR1 protein expression from MI and healthy cell lysates cultured in the 3D system was significantly higher than VEGF protein expression in MI and healthy cell lysates cultured in the 2D system. The expression levels of VEGF protein were severely elevated in conditioned media from the 3D cultures. Control/Healthy cell: cardiomyocytes isolated from healthy NMRI mice. MI cell: cardiomyocytes isolated from myocardial infarction NMRI mice. The values are shown as the means and standard deviation of the mean (mean ± SD). *p < 0.05, **p ≤ 0.01, and ***p ≤ 0.0001 shows the significant change in comparison to control (*p* < 0.05). ^#^shows the significant change in comparison to the MI group (*p* < 0.05)

Western blot results for protein expression of VEGFR1 in cell lysates (Fig. 4A) showed that in healthy cells cultured on an acellular aorta matrix, VEGFR1 protein expression significantly increased compared to the control group (*p* ≤ 0.01). However, in MI cells cultured in the 2D system, VEGFR1 protein expression was significantly decreased compared to control (*p* < 0.0001). Importantly, the data revealed a remarkable elevated expression of VEGFR1 protein in MI cells grown on 3D cultures compared to the MI group (2D system). According to the results shown in Fig. 4B, the level of VEGF in conditioned media from acellular aorta + MI cell and acellular aorta + healthy cell groups was higher than that of collected conditioned media 2D cultures.

### Redox-related factor, MDA, in cardiomyocytes isolated from MI and healthy heart

**Fig. 5:**
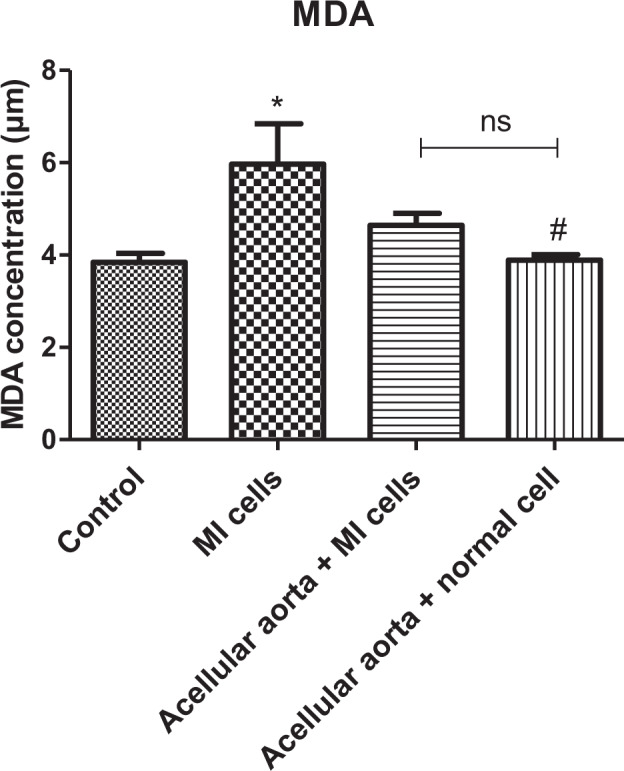
The measurement of the MDA levels in cardiomyocyte isolated from the MI and healthy heart. The MDA levels were increased in the MI group, while the 3D culture system reduced the concentration of MDA compared to the MI group. *shows the significant change in comparison to control (*p* < 0.05). ^#^shows a significant change in comparison to the MI group (*p* < 0.05)

MDA was increased in MI-derived cardiomyocytes cultured as a 2D culture system compared to the control (healthy) cells (*p* ≤ 0.05). Cell cultures in the 3D system showed a reduction in the MDA levels compared to 2D-cultured MI cells. However, the observed alterations of MDA level were only significant (*p* < 0.05) in 3D-cultured healthy cardiomyocytes in comparison to 2D-cultured MI cells. No significant reduction was observed between 2D and 3D-cultured MI cells. Furthermore, there were no significant differences in the MDA level in both 3D-cultured healthy and MI-induced cardiomyocytes comparing to control (healthy) groups (2D system) (Fig. 5).

## Discussion

ECM has been known as an essential member of myocardial healing following ischemic injury and a crucial mediator for cell regeneration and endogenous tissue reconstruction [24–26]. Natural acellular tissue 3D cell culture systems have attracted increasing interest in tissue engineering due to better simulating the microenvironment where in vivo cells grow [27–32].

This study investigated the growth, proliferation, and angiogenesis signaling pathway in cardiomyocytes derived from isoproterenol-induced MI and healthy mice cultured on the acellular aortic matrix (3D system) and compared them with healthy and injured cells cultured in a 2D system. Consequently, as the first step, the acellular matrix was prepared from NMRI mice cardiac aorta, and the growth of cardiomyocytes on it was verified in vitro. Then, real-time PCR and western blotting analysis revealed that the levels of *Hif-1α* and *VEGF* genes expression and VEGFR1 protein expression were considerably upregulated in cardiomyocytes cultured on decellularized myocardial aorta (3D system). Moreover, the level of detected VEGF was significantly high in conditioned media collected from the acellular aorta. The diminished MDA level revealed that oxidative stress is reduced in cardiomyocytes cultured in the 3D system compared to control (2D).

3D culture system led to an accelerated cell proliferation compared to the 2D culture system. After cell seeding, until day 3, cell proliferation was increased in the 3D culturing system but not significantly; afterward, on day 14, a high amount of proliferation was detected. It is well-identified that during cardiomyocytes differentiation, the proliferation rate reduced from progenitor cells to early and matured cardiomyocytes [33, 34]. In line with our results, Fleisher et al. reported that during initiation of differentiation, until day 7, a high amount of proliferating cells was detectable in 3D cultures compared to 2D culture. Various investigations have revealed that in addition to angio/lymphangiogenesis, the VEGF family is involved in other cellular actions such as cell survival. It was demonstrated that VEGF expression leading to VEGFRs activation and downstream signaling pathways to promote cell survival, migration, and angiogenesis [35–37]. Therefore, in addition to biomechanical impacts on the cells, the higher cell viability in cells cultured in the 3D system can be related to the preservation of VEGF in scaffold and VEGF upregulation in cardiomyocytes.

Our in vitro experiments demonstrated a significant difference in angiogenesis ability between cardiomyocytes under 2D and 3D conditions, as the upregulation of *VEGF* and *Hif-1α* gene expression and VEGFR1 protein expression was confirmed. These results suggest that acellular aorta via ECM mimicking and probably using VEGF release induced the MI and healthy cardiomyocytes to express angiogenic markers. On the other hand, VEGF as the most important angiogenic factor is present in our acellular cardiac aorta and so in their collected conditioned media. In line with our results, different studies revealed that native bioactive constituents present in the ECM scaffolds, such as VEGF [9, 10]. In addition, Badylak et al. described acellular biological tissues showing that the decellularization process does not disturb native bioactive components present in the ECM scaffolds, such as FGF-2 and VEGF [38].

Traditionally, VEGF is identified as a critical mediator of angiogenesis, which induces the differentiation, proliferation, and migration of endothelial cells, consequently contributing to the formation of vessels through both angiogenesis and vascular remodeling [39]. It acts through regulating angiogenesis via the hypoxia-inducible factor 1 alpha (HIF-1α)/VEGFA pathway [40]. Hence, as a result, a collected conditioned medium of acellular cardiac aorta cell culture that is containing VEGF can effectively induce cardiac angiogenesis. Thus, it may potentially be used as a cell culture scaffold for cell therapy in post-MI treatment.

MDA is a lipid peroxidation marker used to evaluate lipid peroxidation induced by increased oxidative stress. The increase of oxidative stress in the infarcted myocardium is further verified by oxidative stress markers [41]. It seems that our 3D culture system with the microenvironment simulating reduced oxidative stress in cardiomyocytes and thus increased cell survival compared to the 2D cell culture system.

## Conclusion

In the present study, we demonstrated that AAMs could improve cell viability, preserve VEGF expression, and stimulate the angiogenesis signaling pathways via upregulating the expression of *Hif-1α*, *VEGF* genes, and VEGFR1 protein in cardiomyocytes derived from both normal and MI-induced myocardium tissues of mice. Thus, it seems that these acellular matrices could be considered as a potential approach along with other therapeutic methods to improve cell proliferation and survival of cardiomyocytes in MI-induced hearts, consequently relieving partly the pathological conditions caused by myocardial infarction. However, further studies are needed to confirm the in vivo effects of acellular aorta scaffold for myocardium regeneration.
